# A case series of post-partum haemorrhage managed using Ellavi uterine balloon tamponade in a rural regional hospital

**DOI:** 10.4102/safp.v63i1.5266

**Published:** 2021-05-11

**Authors:** Gerhard B. Theron, Vulikaya Mpumlwana

**Affiliations:** 1 Department of Obstetrics and Gynaecology, Faculty of Medicine and Health Sciences, Stellenbosch University, Cape Town, South Africa; 2 Department of Obstetrics and Gynaecology, Faculty of Health Sciences, Walter Sisulu University, Mthatha, South Africa

**Keywords:** rural regional hospital, post-partum haemorrhage, Ellavi uterine balloon

## Abstract

Uterine balloon tamponade (UBT) should be attempted once emergency measures have been applied and medical treatment for post-partum haemorrhage (PPH) resulting from an atonic uterus has failed. Sinapi Biomedical (Pty) Ltd developed the Ellavi UBT, a free-flow pressure-controlled UBT unit. The device is affordable for use in lesser-resourced countries. A case series of Ellavi UBT used by medical officers in a rural regional hospital without specialist supervision was conducted. This case series was conducted in St Elizabeth’s Hospital in Lusikisiki, South Africa. The hospital serves as the regional hospital for the Ingquza Hill Subdistrict in the Eastern Cape Province. The Nelson Mandela Academic Hospital (NMAH) in Mthatha is the tertiary referral hospital. Workshops were conducted on the use of Ellavi UBT, and devices were made freely available to the hospital. The case series included 10 patients. Six patients delivered by caesarean section, and four had normal vertex deliveries. All patients had additional oxytocin infusions, and eight patients received misoprostol. Following the insertion and inflation of the Ellavi UBT, the PPH stopped in seven patients, was much reduced in one patient and reduced in one patient. In one case, the Ellavi UBT had no effect on the bleeding. All 10 patients were referred to the NMAH. All patients in the case series had good outcomes. The insertion of the Ellavi UBT and subsequent referral proved to be feasible in a rural regional hospital. All patients included in the case series arrived at the referral hospital and had a good outcome.

## Introduction

In South Africa, haemorrhage was the second most important direct cause of maternal deaths in the 2014–2016 triennial report by the National Committee for Confidential Enquiries into Maternal Deaths.^1^ Fawcus^2^ determined the outcomes of women with obstetric haemorrhage that required referral to the next level of care. Obstetric haemorrhage caused 624 deaths, with 128 women requiring referral. Of these women, 25 (20.8%) died whilst waiting for ambulance transport and a further 17 (14.2%) during ambulance transfer. The mean waiting time was 3.7 h, and the range was 1–11 h. An additional measure to reduce blood loss is urgently required when women require referral.

Uterine balloon tamponade (UBT) is one of the steps in the management bundle for post-partum haemorrhage (PPH) and should be attempted once emergency measures have been applied, trauma and retained placental fragments excluded and medical treatment for PPH resulting from an atonic uterus has failed.^3,4,5^ The findings of the most recent systematic review reveal a discrepancy between non-randomised studies and randomised controlled trials of UBT in the treatment of severe PPH.^5^ The need for a large high-quality implementation randomised control trial on the use of UBT remains.

Sinapi Biomedical (Pty) Ltd developed the Ellavi UBT, a free-flow pressure-controlled UBT unit.^6^ The device is affordable for use in lesser-resourced countries.^7^ A case series was conducted to assess the feasibility and acceptance of the Ellavi UBT by physicians in a tertiary hospital and three neighbouring district hospitals.^8^ The use of the Ellavi UBT was feasible and well accepted. The next step was to conduct a case series of Ellavi UBT used by medical officers in a rural regional hospital without specialist supervision to assess the feasibility and acceptance.

## Methods

A case series was conducted in St Elizabeth’s Hospital (SEH) in Lusikisiki. The hospital serves as the regional hospital for the Ingquza Hill Subdistrict of the O.R. Tambo District in the Eastern Cape Province. The Nelson Mandela Academic Hospital (NMAH) in Mthatha is the tertiary referral hospital.

Workshops were conducted by the principal investigator at SHE on the use of the Ellavi UBT. The management bundles for PPH following vaginal delivery and caesarean section (CS) compiled by the National Committee for Confidential Enquiries into Maternal Deaths were explained and discussed. A management brochure with instructions was attached to each sterile-packed Ellavi UBT. Ongoing profuse bleeding following Ellavi UBT insertion and inflation required the next step in the management bundle to be implemented immediately, as well as in the case of bleeding not considerably decreased within 15 min of insertion. Successful use of the Ellavi UBT was considered a reduction in PPH following the uterine balloon insertion and inflation, until ambulance transfer to the NMAH, and maternal survival following transfer. Ellavi UBT devices were made freely available to the hospital.

With PPH, when the clinical requirements for the use of a UBT arose, the contents of the patient informed consent form (ICF) were briefly discussed with the patient and signed. When the patient’s clinical condition stabilised, the patient had the opportunity to read the ICF and ask questions. In the event of the use of a UBT in an operating theatre following a CS or severe shock, clinical notes were made in the patient’s file and the patient informed when awake or stable that an Ellavi UBT had been used.

The projected sample size was 15 patients.

### Ethical considerations

With PPH, when the clinical requirements for the use of UBT arose, the content of the patient informed consent form (ICF) was briefly discussed and signed. When the patient’s clinical condition stabilised, the patient had the opportunity to read the ICF and ask questions. In the event of the use of an UBT in an operating theatre following a caesarean section or severe shock, clinical notes were made in the patient’s file, and the patient was informed that an Ellavi UBT was used. Written informed consent was obtained prior to the interviews.

Approval for the case series was obtained from the Health Research Ethics Committee of the Stellenbosch University (reference number N16/04/044) and the Walter Sisulu University, Faculty of Health Sciences, Postgraduate Education, Training, Research and Ethics Unit (protocol number 032/2018). The protocol is registered with the South African Trial Register.

## Results

A total of four training visits occurred from 08 May 2018 to 13 November 2018. Seven medical officers were trained, including one sessional doctor who did calls for obstetrics. Subsequently, three more visits occurred during 2019.

The case series included 10 patients who had Ellavi UBT insertions. No cases were reported where the Ellavi UBT was taken from the sterile package with intention to use but not used. Summary data were calculated from patients with recorded information. The median age of the patients was 30 years (range: 21–43), and the median parity was 3 (range: 1–6). The median haemoglobin concentration at the first antenatal visit was 11.7 g/dL (range: 10.8 g/dL – 13.5 g/dL). The median weight at the first antenatal visit was 74.5 kg (range: 69 kg – 80 kg). The gestational age at the first antenatal visit was 19 weeks (range: 15–26 weeks). Two patients had two previous CSs, one patient had one previous CS and one a previous fresh stillborn baby. The four women living with human immunodeficiency virus (WLWHIV) were all on highly active antiretroviral therapy (HAART) with World Health Organization Stage 1 disease. Two patients developed pre-eclampsia, one pregnancy-induced hypertension and one went into preterm labour.

Details of the patients who had uterine balloon placements are shown in Table 1. Six patients delivered by CS, including the three patients with previous CSs, and four had normal vertex deliveries. The cause of the PPH was atonic uterus in five patients; in two patients the uterus was well contracted, and in three cases no information was provided. All patients had additional oxytocin infusions; eight patients received misoprostol as an additional oxytocic, and one patient who received misoprostol also received ergometrine. Tranexamic acid was administered to three patients. The blood loss ranged between 800 mL and 2000 mL prior to uterine balloon insertion in four patients with blood loss recorded. The use of blood products was recorded in seven patients. A median of four units of blood was used with a range of three to six units. Two patients received fresh frozen plasma and one patient cryoprecipitate.

**TABLE 1:** Details of patients who had uterine balloon placements.

Case	GA at delivery (weeks)	Mode of delivery	Atonic uterus (cause of PPH)	Additional oxytocic(s) used	Tranexamic acid	Blood loss volume (ml) prior to UBT	Compression sutures prior to UBT	Duration balloon in uterus (h:min)	Additional surgery following UBT (location)
1	40	CS	Yes	Misoprostol	Yes	2000	B-Lynch	-	TAH (NMAH)
2	37	CS	No	Misoprostol	Yes	-	Laparotomy	-	-
3	40	CS	Yes	Misoprostol + ergometrine	-	-	-	-	Subtotal AH (SEH)
4	-	NVD	Yes	-	-	-	-	12:00	-
5	37	NVD	-	Misoprostol	-	800	-	7:00	TAH (NMAH)
6	32	CS	No	Misoprostol	Yes	-	-	6:00	-
7	39	NVD	Yes	Misoprostol	-	1000	-	-	-
8	35	CS	-	Misoprostol	-	2000	Hayman	4:00	-
9	-	NVD	-	-	-	-	-	2:30	TAH (NMAH)
10	-	CS	Yes	Misoprostol	-	-	B-Lynch	-	-

GA, gestational age; PPH, post-partum haemorrhage; NVD, normal vertex delivery; CS, caesarean section; UBT, uterine balloon tamponade; TAH, total abdominal hysterectomy; AH, abdominal hysterectomy; NMAH, Nelson Mandela Academic Hospital; SHE, St Elizabeth’s Hospital.

B-Lynch compression sutures were inserted in two patients and a Hayman compression suture in one patient prior to uterine balloon insertion. One patient had a laparotomy prior to uterine balloon insertion without the use of compression sutures. Following insertion and inflation of the Ellavi UBT, the PPH stopped in seven patients, was much reduced in one patient and reduced in one patient. In one case, the Ellavi UBT had no effect on the bleeding. The duration for which the uterine balloons remained in the uterus ranged from 2 h 30 min to 12 h in the five patients with available information.

All 10 patients were referred to the NMAH. Additional surgery was done following the uterine balloon insertion in four patients. One patient had a subtotal abdominal hysterectomy in SEH, and three patients had total abdominal hysterectomies subsequently to referral from SEH.

The one unsuccessful case was a 22-year-old gravida 2 para 1 with a previous normal vertex delivery (Case 3, Table 1). She is a WLWHIV and on HAART. Her viral load was lower than detectable. She had a CS following poor progress in the first stage of labour. The duration of her first stage of labour was 10 h. Following the CS, she had bleeding from an atonic uterus. Misoprostol and ergometrine were administered in addition to the oxytocin infusion. The Ellavi UBT was inserted and inflated but did not reduce the bleeding. An emergency subtotal hysterectomy was performed without delay. Six units of blood and three units of fresh frozen plasma were infused. She also received tranexamic acid. She was referred to the NMAH, where she was kept in a high care unit for two days and discharged in good health.

Physician questionnaires were completed by the seven physicians who did Ellavi UBT insertions. The placement was regarded as easy in six patients and fairly easy in one case. All placements were made by hand only, and in two cases a swab-holding forceps was placed on the cervix. Filling the uterine balloon from the supply bag or with intravenous fluid and managing intra-uterine pressure according to the systolic blood pressure did not pose any problems. The Ellavi UBT stopped the bleeding in 9 out of 10 patients. No complications (i.e. uterine perforations or infectious complications) resulted from the use of the Ellavi UBT, and all patients in the case series had a good outcome.

During the focus group discussion, all seven doctors were in favour of a pre-assembled sterile device as a single unit instead of a makeshift device that needs to be assembled. They did not experience problems with inserting or filling the uterine balloon. None of the patients experienced abdominal discomfort following filling of the uterine balloon. None of the nine patients transferred with uterine balloons were expelled prior to departure from SEH. The answers provided during the focus group discussion regarding the unique features of the Ellavi UBT included that it is simple to use and that uterine placement is easier compared to a condom used as a uterine balloon. The physicians found that the uterine balloon fills quickly, and a rapid assessment can be made as to whether the UBT stops or reduces the bleeding. They stated that the Ellavi UBT can be safely used together with compression sutures. The group were impressed by the success rate of stopping PPH with the use of the Ellavi UBT, and all were of the opinion that Ellavi UBTs should be available in all birthing facilities and hospitals. The obstetrics nursing unit manager in the labour ward did confirm that no maternal deaths resulting from PPH occurred during the study period.

## Discussion

The aim with the treatment of refractory PPH at SEH is to stabilise patients and transfer them to the NMAH, as additional surgical management at SEH is limited. Examination under anaesthetics, evacuation of the uterus and compression sutures could be done by all the medical officers, and one of the medical officers was trained to do a subtotal hysterectomy. All 10 patients managed at SEH were referred to the NMAH. The distance between SEH and the NMAH is 130 km, and the driving time is 2 h 15 min.

Ellavi UBT insertion followed CS and the use of compression sutures in three patients. Combining external compression with internal tamponade is most effective in arresting the bleeding.^9,10^ Further surgical measures to stop bleeding can be averted at a time when patients have already lost a considerable amount of blood and when clotting deficiencies may be present. The Ellavi UBT allows intrauterine pressure control and can be used safely with compression sutures, avoiding the possibility of myometrial pressure necrosis.^11,12^

The aim of the study was to assess the acceptance and feasibility of the Ellavi UBT by medical officers working without specialist supervision in a busy regional hospital. They were unanimous in welcoming a pre-assembled sterile device as a single unit instead of a makeshift device. The placement was regarded as easy. Filling the uterine balloon and managing intra-uterine pressure according to the systolic blood pressure did not pose any problems, and the concept was well accepted. The time to fill the uterine balloon is much shorter compared to filling a uterine balloon or condom with a 20-mL or 50-mL syringe.^13,14,15^ The short filling time allows for a rapid assessment of the effect of the UBT on the bleeding.

Limitations of the study include the sample size (the target of 15 patients was not reached) and missing clinical information. The time required to include 10 cases was 17 months. Conducting the study over a long distance, staff turnover at SEH and little experience in participating in research contributed to this outcome. The possibility that patients with refractory PPH were managed without the use of a UBT and that some cases were not captured needs to be considered.

## Conclusion

The insertion of the Ellavi UBT and the subsequent management proved to be feasible in a rural regional hospital without specialist supervision, as well as a potentially useful temporising intervention for transfer. All patients included in the case series arrived at the referral hospital and had good outcomes.

## References

[1] Saving Mothers 2014–2016. The seventh report of the National Committee for Confidential Enquiry into Maternal Deaths in South Africa. Short report. Pretoria: Department Health; 2017.

[2] Fawcus S. A focus on referral problems in women who died from obstetric haemorrhage in South Africa (2016–2016). O&G Forum. 2018;28:23–27.

[3] Doumouchtsis SK, Papageorghiou AT, Arulkumaran S. Systematic review of conservative management of postpartum haemorrhage: What to do when medical treatment fails. Obstet Gynaecol Survey. 2007;62(8):540–547. 10.1097/01.ogx.0000271137.81361.93 17634155

[4] Georgiou C. Uterine tamponade in the management of postpartum haemorrhage: A review. BJOG. 2009;116:748–757. 10.1111/j.1471-0528.2009.02113.x 19432563

[5] Suarez S, Conde-Agudelo A, Borovac-Pinheiro A, et al. Uterine balloon tamponade for the treatment of postpartum haemorrhage: A systematic review and meta-analysis. Am J Obstet Gynecol. 2020;222(4):293.e1–52. 10.1016/j.ajog.2019.11.1287 31917139

[6] Theron GB. Uerine balloon tamponade. O&G Forum. 2019;29:1.

[7] Ayres-de-Campos D, Stones W, Theron GB. Affordable and low-maintenance obstetric devices. Int J Gynecol Obstet. 2019;146:25–28. 10.1002/ijgo.12838 31055829

[8] Theron GB. Management of postpartum haemorrhage with free-flow pressure controlled uterine balloon. Int J Gynecol Obstet. 2018;142:371–373. 10.1002/ijgo.12533 29779208

[9] Danso D, Reginald P. Combined B-lynch suture with intrauterine balloon catheter triumphs over massive postpartum haemorrhage. BJOG. 2002;109:693. 10.1111/j.1471-0528.2002.01111.x 12197382

[10] Diemert A, Ortmeyer G, Hollwitz B, et al. The combination of intrauterine balloon tamponade and the B-Lynch procedure for the treatment of severe postpartum hemorrhage. Am J Gynecol. 2012;206(1):65e1–4. 10.1016/j.ajog.2011.07.041 22000893

[11] Treloar EJ, Anderson RS, Andrews HS, Bailey JL. Uterine necrosis following B-Lynch sutures for primary postpartum haemorrhage. BJOG. 2006;113(4):486–488. 10.1111/j.1471-0528.2006.00890.x 16553658

[12] Lodhi W, Golara M, Karagoakar V, Yoong W. Uterine necrosis following application of combined uterine compression suture with intrauterine balloon tamponade. J Obstet Gynaecol. 2012;32(1):30–31. 10.3109/01443615.2011.614972 22185530

[13] Nelson BD, Stoklosa H, Ahn R, Eckardt MJ, Walton EK, Burke TF. Use of uterine balloon tamponade for control of postpartum haemorrhage by community-based health providers in South Sudan. Int J Gynecol Ostet. 2013;122:27–32. 10.1016/j.ijgo.2013.02.017 23623587

[14] Mishra N, Agrawal S, Gulabani K, Shrivastava C. Use of an innovative condom balloon tamponade in postpartum haemorrhage: A report. J Obstet Gynecol India. 2016;66:63–67. 10.1007/s13224-015-0818-2 26924911 PMC4755948

[15] Dumont A, Bodin C, Hounkpatin B, et al. Uterine balloon tamponade as an adjunct to misoprostol for the treatment of uncontrolled postpartum haemorrhage: A randomised controlled trial in Benin and Mali. BMJ Open 2017;7(9):e016590. 10.1136/bmjopen-2017-016590 PMC5589006 28864699

